# Supplementary material for: Serological surveillance reveals a high exposure to SARS-CoV-2 and altered immune response among COVID-19 unvaccinated Cameroonian individuals
Source: PLOS Glob Public Health. 2024 Feb 12;4(2):e0002380. doi: 10.1371/journal.pgph.0002380 (PMC10861046; doi:10.1371/journal.pgph.0002380)

**S1 Fig**. **Ethical clearance of the study: English translation and original clearance (French).**

**English translation**


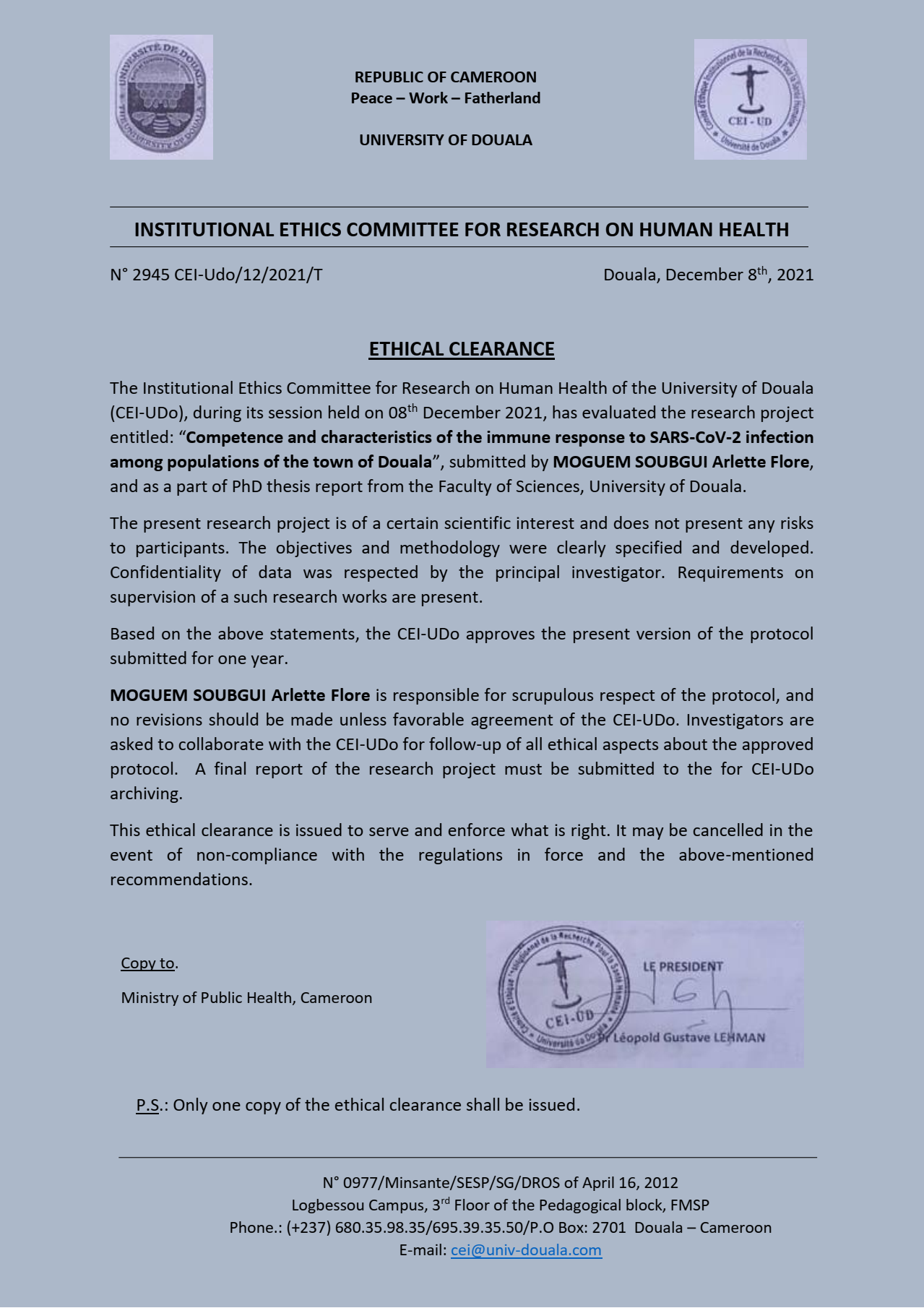


**Original clearance (French)**


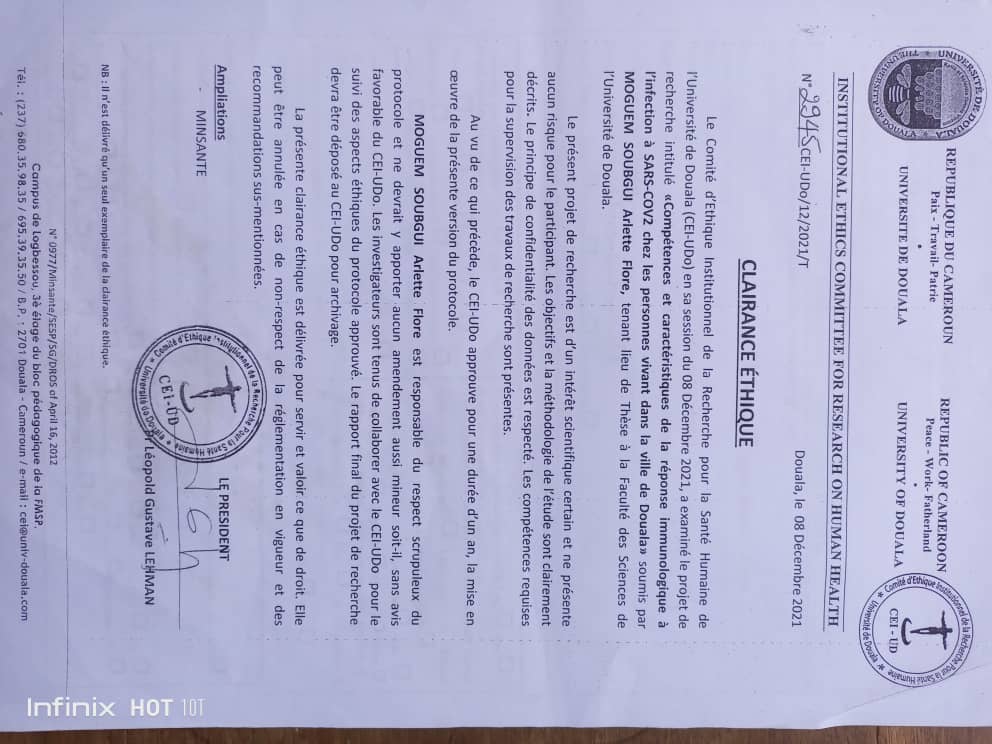

Supplement: S1 Fig — (DOCX) [file pgph.0002380.s001.docx]
