# Supplementary material for: Serological surveillance reveals a high exposure to SARS-CoV-2 and altered immune response among COVID-19 unvaccinated Cameroonian individuals
Source: PLOS Glob Public Health. 2024 Feb 12;4(2):e0002380. doi: 10.1371/journal.pgph.0002380 (PMC10861046; doi:10.1371/journal.pgph.0002380)

**S2 Fig. Testing SARS-CoV-2 immune response parameters (IgM, IgG, CD4+, IFN-γ and IL-6) and age for Gaussian distribution.**

1. **IgM**

| **Statistics** | | |
| --- | --- | --- |
| anti IgM index | |  |
| N | Valid | 342 |
|  | Missing | 0 |
| Mean | | 1.722391E0 |
| Median | | .942000 |
| Mode | | .0010 |
| Skewness | | 2.069 |
| Std. Error of Skewness | | .132 |
| Kurtosis | | 4.545 |
| Std. Error of Kurtosis | | .263 |


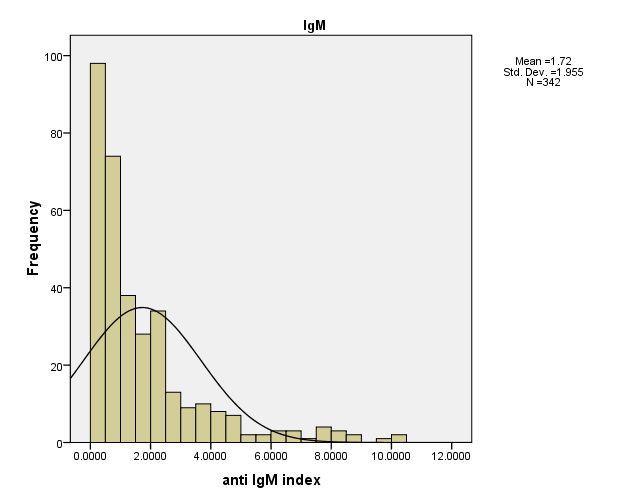


1. **IgG**

| **Statistics** | | |
| --- | --- | --- |
| anti IgG index | |  |
| N | Valid | 341 |
|  | Missing | 1 |
| Mean | | 6.5748 |
| Median | | 4.8300 |
| Mode | | 1.48^a^ |
| Skewness | | 1.019 |
| Std. Error of Skewness | | .132 |
| Kurtosis | | .162 |
| Std. Error of Kurtosis | | .263 |
| a. Multiple modes exist. The smallest value is shown | | |


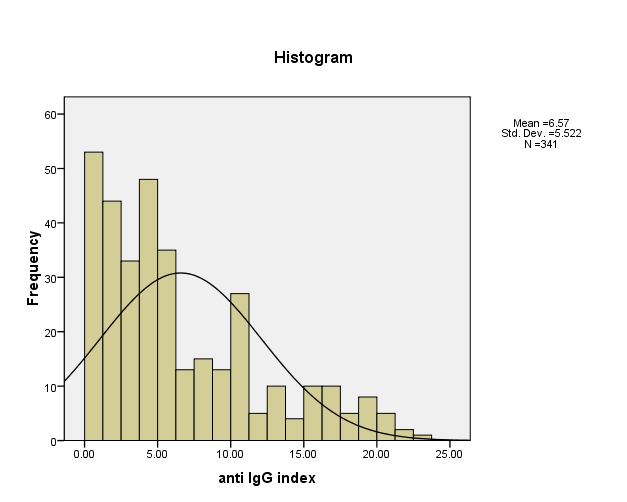


1. **CD4+**

| **Statistics** | | |
| --- | --- | --- |
| CD4 |  |  |
| N | Valid | 145 |
|  | Missing | 197 |
| Mean | | 557.32 |
| Median | | 552.00 |
| Mode | | 470 |
| Skewness | | 1.041 |
| Std. Error of Skewness | | .201 |
| Kurtosis | | 3.823 |
| Std. Error of Kurtosis | | .400 |


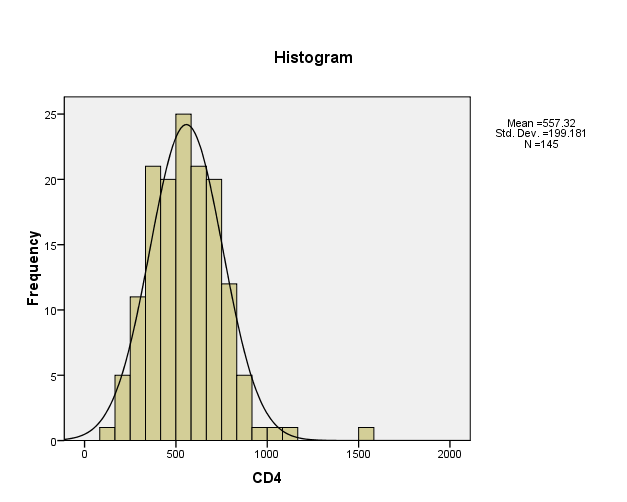


1. **IFN-gamma**

| **Statistics** | | |
| --- | --- | --- |
| INF-gamma | |  |
| N | Valid | 129 |
|  | Missing | 213 |
| Mean | | .335890 |
| Median | | .203000 |
| Mode | | .1750 |
| Skewness | | 3.056 |
| Std. Error of Skewness | | .213 |
| Kurtosis | | 9.713 |
| Std. Error of Kurtosis | | .423 |


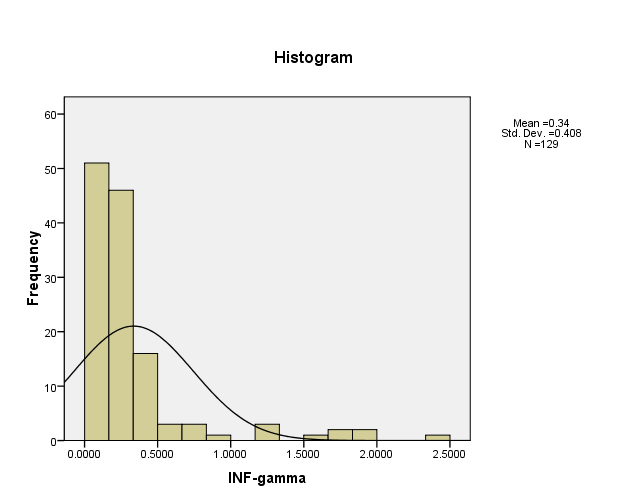


1. **IL-6**

| **Statistics** | | |
| --- | --- | --- |
| IL-6 |  |  |
| N | Valid | 132 |
|  | Missing | 210 |
| Mean | | 2.84215E1 |
| Median | | 7.64300 |
| Mode | | .021 |
| Skewness | | 7.336 |
| Std. Error of Skewness | | .211 |
| Kurtosis | | 67.395 |
| Std. Error of Kurtosis | | .419 |


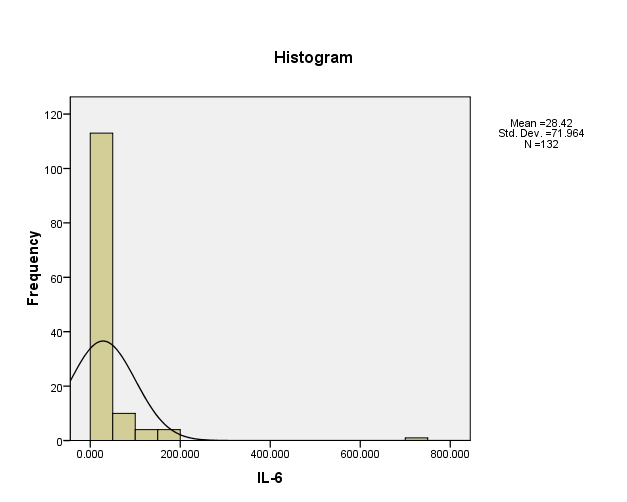


1. **Age**

| **Statistics** | | |
| --- | --- | --- |
| Age |  |  |
| N | Valid | 342 |
|  | Missing | 0 |
| Mean | | 41.48 |
| Median | | 38.00 |
| Mode | | 27 |
| Skewness | | .612 |
| Std. Error of Skewness | | .132 |
| Kurtosis | | -.448 |
| Std. Error of Kurtosis | | .263 |


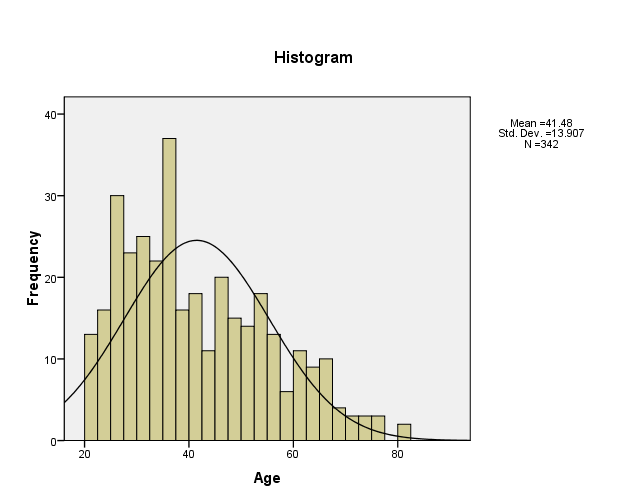

Supplement: S2 Fig — (DOCX) [file pgph.0002380.s002.docx]
