# Supplementary material for: Serological surveillance reveals a high exposure to SARS-CoV-2 and altered immune response among COVID-19 unvaccinated Cameroonian individuals
Source: PLOS Glob Public Health. 2024 Feb 12;4(2):e0002380. doi: 10.1371/journal.pgph.0002380 (PMC10861046; doi:10.1371/journal.pgph.0002380)
Supplement: S1 Table — (DOCX) [file pgph.0002380.s003.docx]

**S1 Table. Crude SARS-CoV-2 seroprevalence profile** **by months and health facilities.**

|  |  | **IgM** | | |  | **IgG** | | |  | **IgM + IgG** | | |
| --- | --- | --- | --- | --- | --- | --- | --- | --- | --- | --- | --- | --- |
| **Variables** | **N** | ***n*** | **%** | **95%CI** |  | ***n*** | **%** | **95%CI** |  | ***n*** | **%** | **95%CI** |
| **Months** |  |  |  |  |  |  |  |  |  |  |  |  |
| January | **22** | 12 | 54.5 | 34.7 - 67.1 |  | 20 | 90.9 | 72.2 - 97.5 |  | 22 | 100.0 | 85.1 - 100 |
| February | **36** | 16 | 44.4 | 29.5 - 60.4 |  | 32 | 88.9 | 74.7 - 95.6 |  | 36 | 100.0 | 90.3 - 100 |
| March | **132** | 62 | 47.0 | 38.7 - 55.5 |  | 114 | 86.4 | 79.5 - 91.7 |  | 127 | 96.2 | 91.7 - 98.4 |
| April | **50** | 31 | 62.0 | 48.2 - 74.1 |  | 43 | 86.0 | 73.8 - 93.1 |  | 49 | 98.0 | 89.5 - 99.7 |
| May | **16** | 4 | 25.0 | 10.2 - 49.5 |  | 15 | 93.8 | 71.7 - 98.9 |  | 15 | 93.8 | 71.7 - 98.9 |
| June | **29** | 15 | 51.7 | 34.4 - 68.6 |  | 28 | 96.6 | 82.8 - 99.4 |  | 28 | 96.6 | 82.8 - 99.4 |
| July | **26** | 18 | 69.2 | 50.0 - 83.1 |  | 24 | 92.3 | 75.9 - 97.9 |  | 26 | 100.0 | 75.9 - 97.9 |
| August | **21** | 7 | 33.3 | 17.2 - 54.6 |  | 18 | 85.7 | 65.4 - 95.0 |  | 18 | 85.7 | 65.4 - 95.0 |
| September | **10** | 3 | 30.0 | 10.8 - 60.3 |  | 10 | 100.0 | 72.3 - 100 |  | 10 | 100.0 | 72.3 - 100 |
| **χ^2^ (df)** |  | 15.71 (8) | | |  | 5.24 (8) | | |  | 12.24 (8) | | |
| ***p*-value** |  | **0.04*** | | |  | 0.73 | | |  | 0.14 | | |
| **Health facilities** |  |  |  |  |  |  |  |  |  |  |  |  |
| Bangue | **108** | 54 | 50.0 | 40.7 - 59.3 |  | 99 | 91.7 | 84.9 - 95.6 |  | 104 | 96.3 | 90.9 - 98.6 |
| Boko | **19** | 8 | 42.1 | 23.2 - 63.7 |  | 19 | 100 | 83.2 - 100 |  | 19 | 100 | 83.2 - 100 |
| Bonassama | **62** | 36 | 58.1 | 45.7 - 69.5 |  | 56 | 90.3 | 80.5 - 95.5 |  | 62 | 100 | 94.2 - 100 |
| Cité des Palmiers | **63** | 33 | 52.4 | 40.3 - 63.2 |  | 56 | 88.9 | 78.8 - 94.5 |  | 60 | 95.2 | 86.9 - 98.4 |
| Deido | **22** | 10 | 45.5 | 26.9 - 65.3 |  | 14 | 63.6 | 49.2 - 80.3 |  | 20 | 90.9 | 72.2 - 97.5 |
| New-Bell | **39** | 16 | 41.1 | 27.1 - 56.6 |  | 36 | 92.3 | 79.7 - 97.4 |  | 39 | 100 | 91.0 - 100 |
| Nylon | **29** | 11 | 37.9 | 22.7 - 56.0 |  | 24 | 82.8 | 65.5 - 92.4 |  | 27 | 93.1 | 78.0 - 98.1 |
| **χ^2^ (df)** |  | 5.25 (6) | | |  | 19.12 (6) | | |  | 8.25 (6) | | |
| ***p*-value** |  | 0.51 | | |  | **0.004*** | | |  | 0.22 | | |

95%CI: Confidence interval at 95%, df: Degree of freedom, Ig: Immunoglobulin, SARS–CoV–2: Severe acute respiratory syndrome coronavirus 2

Data are presented frequency (*n*) and percentages (%)

Pearson’s independence chi-square test was used to compare percentages

*Statistically significant at *p*-value < 0.05
