# Supplementary material for: Serological surveillance reveals a high exposure to SARS-CoV-2 and altered immune response among COVID-19 unvaccinated Cameroonian individuals
Source: PLOS Glob Public Health. 2024 Feb 12;4(2):e0002380. doi: 10.1371/journal.pgph.0002380 (PMC10861046; doi:10.1371/journal.pgph.0002380)
Supplement: S2 Table — (DOCX) [file pgph.0002380.s004.docx]

**S2 Table. Crude SARS-CoV-2 seroprevalence profile by clinical symptoms.**

|  |  |  | **IgM** | |  | **IgG** | |  | **IgG + IgM** | |
| --- | --- | --- | --- | --- | --- | --- | --- | --- | --- | --- |
| **Symptoms** | **Categories** | **N** | *n* | % |  | *n* | % |  | *n* | % |
| **Cough** | No | **293** | 139 | 47.4 |  | 271 | 92.5 |  | 284 | 96.9 |
|  | Yes | **49** | 29 | 59.2 |  | 33 | 67.3 |  | 47 | 95.9 |
|  | *p*-value |  | 0.16 | |  | **< 0.0001*** | |  | 0.66 | |
| **Severe fatigue** | No | **302** | 139 | 46.1 |  | 277 | 91.7 |  | 292 | 96.7 |
|  | Yes | **40** | 29 | 72.5 |  | 27 | 67.5 |  | 39 | 97.5 |
|  | *p*-value |  | **0.002*** | |  | **0.001*** | |  | 1 | |
| **Headache** | No | **307** | 142 | 46.3 |  | 276 | 89.9 |  | 296 | 96.4 |
|  | Yes | **35** | 26 | 74.3 |  | 28 | 80 |  | 35 | 100 |
|  | *p*-value |  | **0.002*** | |  | 0.08 | |  | 0.61 | |
| **Fever** | No | **310** | 145 | 46.8 |  | 284 | 91.6 |  | 300 | 96.8 |
|  | Yes | **32** | 23 | 71.9 |  | 20 | 62.5 |  | 31 | 96.9 |
|  | *p*-value |  | **0.008*** | |  | **< 0.0001*** | |  | 1 | |
| **Respiratory distress** | No | **315** | 149 | 47.3 |  | 284 | 90.2 |  | 304 | 96.5 |
|  | Yes | **27** | 19 | 70.4 |  | 20 | 74.1 |  | 27 | 100 |
|  | *p*-value |  | **0.02*** | |  | **0.01*** | |  | 1 | |
| **Sore throat** | No | **315** | 151 | 47.9 |  | 282 | 89.5 |  | 304 | 96.5 |
|  | Yes | **27** | 17 | 63 |  | 22 | 81.5 |  | 27 | 100 |
|  | *p*-value |  | 0.16 | |  | 0.21 | |  | 1 | |
| **Running nose** | No | **319** | 152 | 47.6 |  | 288 | 90.3 |  | 308 | 96.6 |
|  | Yes | **23** | 16 | 69.6 |  | 16 | 69.6 |  | 23 | 100 |
|  | *p*-value |  | 0.05 | |  | **0.007*** | |  | 1 | |
| **Ageusia** | No | **320** | 148 | 46.3 |  | 292 | 91.3 |  | 309 | 96.6 |
|  | Yes | **22** | 20 | 90.9 |  | 12 | 54.5 |  | 22 | 100 |
|  | *p*-value |  | **< 0.0001*** | |  | **< 0.0001*** | |  | 1 | |
| **Anosmia** | No | **325** | 154 | 47.4 |  | 294 | 90.5 |  | 314 | 96.6 |
|  | Yes | **17** | 14 | 82.4 |  | 10 | 58.8 |  | 17 | 100 |
|  | *p*-value |  | **0.005*** | |  | **0.001*** | |  | 1 | |

Ig: Immunoglobulin, SARS–CoV–2: Severe acute respiratory syndrome coronavirus 2

Data are presented frequency (*n*) and percentages (%)

Pearson’s independence chi-square and Fisher’s exact tests were used to compare percentages

*Statistically significant at *p*-value < 0.05
