# Supplementary material for: Serological surveillance reveals a high exposure to SARS-CoV-2 and altered immune response among COVID-19 unvaccinated Cameroonian individuals
Source: PLOS Glob Public Health. 2024 Feb 12;4(2):e0002380. doi: 10.1371/journal.pgph.0002380 (PMC10861046; doi:10.1371/journal.pgph.0002380)
Supplement: S3 Table — (DOCX) [file pgph.0002380.s005.docx]

**S3 Table. Clinical value of symptoms for prognostic of IgM seropositivity.**

|  | **Sensitivity** | |  | **Specificity** | |  | **Positive predictive value** | | |  | | **Negative predictive value** | | |
| --- | --- | --- | --- | --- | --- | --- | --- | --- | --- | --- | --- | --- | --- | --- |
| **Symptoms** | n/N | % (95%CI) |  | n/N | % (95%CI) |  | n/N | % (95%CI) |  | | n/N | | % (95%CI) |  |
| Cough | 29/168 | 17.3% (12.3 - 23.7%) |  | 154/174 | 88.5% (82.9 - 92.4%) |  | 29/49 | 59.2% (45.2 - 71.8%) |  | | 154/293 | | 52.6% (46.9 - 58.2%) |  |
| Severe fatigue | 29/168 | 17.3% (12.3 - 23.7%) |  | 163/174 | 93.7% (89.0 - 96.4%) |  | 29/40 | 72.5% (57.2 - 83.9%) |  | | 163/302 | | 53.9% (48.3 - 59.5%) |  |
| Headache | 26/168 | 15.5% (10.8 - 21.7%) |  | 165/174 | 94.8% (90.5 - 97.3%) |  | 26/35 | 74.3% (57.9 - 85.8%) |  | | 166/307 | | 54.1% (48.5 - 59.6%) |  |
| Fever | 23/168 | 13.7% (9.3 - 19.7%) |  | 165/174 | 94.8% (90.5 - 97.3%) |  | 23/32 | 71.9% (54.6 - 84.4%) |  | | 165/310 | | 53.2% (47.7 - 58.7%) |  |
| Respiratory distress | 19/168 | 11.3% (7.4 - 16.9%) |  | 166/174 | 95.4% (91.2 - 97.7%) |  | 19/27 | 70.4% (51.5 - 84.2%) |  | | 166/315 | | 52.7% (47.2 - 58.2%) |  |
| Sore throat | 17/168 | 10.1% (6.4 - 15.6%) |  | 164/174 | 94.3% (89.7 - 96.9%) |  | 17/27 | 62.9% (44.2 - 78.5%) |  | | 164/315 | | 52.1% (46.6 - 57.5%) |  |
| Running nose | 16/168 | 9.5% (5.9 - 14.9%) |  | 167/174 | 95.9% (91.9 - 98.0%) |  | 16/23 | 69.6% (49.1 - 84.4%) |  | | 167/319 | | 52.4% (46.9 - 57.8%) |  |
| Ageusia | 20/168 | 11.9% (7.8 - 17.7%) |  | 172/174 | 98.9% (95.9 - 99.7%) |  | 20/22 | 90.9% (72.2 - 97.5%) |  | | 172/320 | | 53.8% (48.3 - 59.1%) |  |
| Anosmia | 14/168 | 8.3% (5.0 - 13.5%) |  | 171/174 | 98.3% (95.2 - 99.4%) |  | 14/17 | 82.4% (58.9 - 93.8%) |  | | 171/325 | | 52.6% (47.2 - 57.9%) |  |

95%CI: Confidence interval at 95%
