# Supplementary material for: Serological surveillance reveals a high exposure to SARS-CoV-2 and altered immune response among COVID-19 unvaccinated Cameroonian individuals
Source: PLOS Glob Public Health. 2024 Feb 12;4(2):e0002380. doi: 10.1371/journal.pgph.0002380 (PMC10861046; doi:10.1371/journal.pgph.0002380)
Supplement: S4 Table — (DOCX) [file pgph.0002380.s006.docx]

**S4 Table. Association between immune response and patients’ characteristics among COVID-19 unvaccinated patients.**

|  |  |  | **IgM** | **IgG** | **IgM + IgG** |
| --- | --- | --- | --- | --- | --- |
| **Variables** | **Categories** | **N** | ***n* (%)** | ***n* (%)** | ***n* (%)** |
| **Age (years)** | < 30 | 82 | 49 (59.8%) | 71 (86.6%) | 78 (95.1%) |
|  | [30 - 40[ | 100 | 46 (46.0%) | 90 (90.0%) | 97 (97.0%) |
|  | [40 - 50[ | 64 | 30 (46.9%) | 59 (92.2%) | 63 (98.4%) |
|  | [50 - 60[ | 51 | 28 (54.9%) | 46 (90.2%) | 51 (100%) |
|  | [60 - 70[ | 34 | 12 (35.3%) | 29 (85.3%) | 32 (94.1%) |
|  | 70+ | 11 | 3 (27.3%) | 9 (81.8%) | 10 (90.9%) |
|  | ***p*-value** |  | **0.04*** | 0.79 | 0.42 |
| **Gender** | Females | 163 | 86 (52.%) | 148 (90.8%) | 158 (96.9%) |
|  | Males | 179 | 82 (45.8%) | 156 (87.2%) | 173 (96.6%) |
|  | ***p*-value** |  | 0.23 | 0.31 | 0.88 |
| **Marital status** | Single | 150 | 82 (54.7%) | 138 (92.0%) | 146 (97.3%) |
|  | Married | 179 | 83 (46.4%) | 154 (86.0%) | 173 (96.6%) |
|  | Divorced/Widow | 13 | 3 (23.1%) | 12 (92.3%) | 12 (92.3%) |
|  | ***p*-value** |  | 0.05 | 0.21 | 0.61 |
| **Educational level** | None/Primary | 22 | 11 (50.0%) | 20 (90.9%) | 21 (95.5%) |
|  | Secondary | 112 | 54 (48.2%) | 100 (89.3%) | 110 (98.2%) |
|  | University | 208 | 103 (49.5%) | 184 (88.5%) | 200 (96.2%) |
|  | ***p*-value** |  | 0.97 | 0.93 | 0.57 |
| **Occupation** | Student | 41 | 26 (63.4%) | 38 (92.7%) | 41 (100%) |
|  | Formal sector | 232 | 107 (46.1%) | 206 (88.9%) | 224 (96.6%) |
|  | Informal sector | 69 | 35 (50.7%) | 60 (87.0%) | 66 (95.7%) |
|  | ***p*-value** |  | 0.12 | 0.65 | 0.43 |
| **Obesity** | No | 258 | 132 (51.2%) | 230 (89.1%) | 251 (97.3%) |
|  | Yes | 84 | 36 (42.9%) | 74 (88.1%) | 80 (95.2%) |
|  | ***p*-value** |  | 0.21 | 0.84 | 0.47 |
| **Diabetes** | No | 321 | 161 (50.2%) | 286 (89.1%) | 311 (96.9%) |
|  | Yes | 21 | 7 (33.3%) | 18 (85.7%) | 20 (95.2%) |
|  | ***p*-value** |  | 0.18 | 0.72 | 0.51 |
| **Hypertension** | No | 305 | 151 (49.5%) | 273 (89.5%) | 296 (97.0%) |
|  | Yes | 37 | 17 (45.9%) | 31 (83.8%) | 35 (94.6%) |
|  | ***p*-value** |  | 0.73 | 0.28 | 0.34 |
| **Heart failure** | No | 334 | 166 (49.7%) | 296 (88.6%) | 323 (96.7%) |
|  | Yes | 8 | 2 (25.0%) | 8 (100%) | 8 (100%) |
|  | ***p*-value** |  | 0.2 | 0.61 | 1 |
| **HIV** | No | 337 | 166 (49.3%) | 300 (89.0%) | 326 (96.7%) |
|  | Yes | 5 | 2 (40.0%) | 4 (40.0%) | 5 (100%) |
|  | ***p*-value** |  | 1 | 0.45 | 1 |
| **Asthma** | No | 331 | 161 (48.6%) | 297 (89.7%) | 320 (96.7%) |
|  | Yes | 11 | 7 (63.6%) | 7 (63.6%) | 11 (100%) |
|  | ***p*-value** |  | 0.37 | **0.02*** | 1 |
| **History of COVID-19** | No | 301 | 150 (49.8%) | 264 (87.7%) | 290 (96.3%) |
|  | Yes | 41 | 18 (43.9%) | 40 (97.6%) | 41 (100%) |
|  | ***p*-value** |  | 0.51 | 0.06 | 0.37 |

COVID-19: Coronavirus disease 2019, HIV: Human immunodeficiency virus infection, Ig: Immunoglobulin

Data are presented frequency (*n*) and percentages (%)

Pearson’s independence chi-square and Fisher’s exact tests were used to compare percentages

*Statistically significant at *p*-value < 0.05
