# Supplementary material for: Serological surveillance reveals a high exposure to SARS-CoV-2 and altered immune response among COVID-19 unvaccinated Cameroonian individuals
Source: PLOS Glob Public Health. 2024 Feb 12;4(2):e0002380. doi: 10.1371/journal.pgph.0002380 (PMC10861046; doi:10.1371/journal.pgph.0002380)
Supplement: Attachment — Submitted filename: Response to Reviewers.docx [file pgph.0002380.s007.docx]

**Response to Reviewers**

**Date: 11.12.2023**

**Manuscript title**: Serological surveillance reveals a high exposure to SARS-CoV-2 and altered immune response among COVID-19 unvaccinated Cameroonian individuals

**Manuscript ID**: PGPH-D-23-01626

**Authors**: Arlette Flore Moguem Soubgui *et al*.

**Journal**: PLOS Global Public Health

**Article type**: Research Article

Dear Journal Editorial Office,

In this letter, we try to respond to each point raised by the reviewers.

We thank the editor and reviewers for their comments about the paper. We are very thrilled with the revised version (R1) of the paper.

Please, find below the answers (brown color) to each of the points raised by reviewers. Revisions are highlighted in the revised manuscript with track changes.

**Reviewer Comments**:

**Reviewer #1**: This is an interesting study. Could you please correct or explain the following comments?

-Page8, Line 96: Information missed:
How were the participants recruited;

**Reply**: Done (Lines 104 - 106).

What brought them to the selected health centers,

**Reply**: We added some lines to explain it (Lines 98 - 101).

Why did you specifically choose these sites,

**Reply**: Please refer to our previous comment (Lines 98 - 101).

How did you approach the patients and suggest they take part in the study?

**Reply**: We added this aspect (Lines 104 - 106).

-Page 9, Line 121: Your study targeted only unvaccinated people. You need to better describe the approach, especially since the collection form (...Vaccination uptake...) mentions vaccination status and this is not one of the non-inclusion criteria.

**Reply**: Dear colleague, we understand your confusion. Even not primarily focused on COVID-19 vaccination, we first presented results on COVID-19 vaccination status of the participants on Figure 2. Then, we excluded vaccinated patients as per the objectives of the study. We added more details to clarity this (Lines 135 - 138). We also added the COVID-19 vaccination status as exclusion criterium (Line 120).

-Page 10, line 132: Precision about the kit used for these tests missed

**Reply**: Corrected (Lines 145 - 149).

-Page 10, Line141: How did you deal with those who didn't understand either of these 2 languages? Especially since that wasn't one of your criteria for non-inclusion.

**Reply**: Since the study was conducted in urban setting, populations of Douala are majorly French-speaking persons, but some are also English-speaking native. In addition, we did not encounter participants not speaking either of these languages. You can face this problem in rural setting. Thus, language was not a non-inclusion criterium in this study.

-Page 13, Line 193: Eliminate the dot at the end of table titles (check all)

**Reply**: Done.

-Page 25, Line355: To compare data with this country, you have to take into account the period, was it the same? Peak period of COVID infection? (Lines 355-357)

**Reply**: We taken into account these different explanations as each COVID-19 vague was due to different virus variants, and in populations with differential risk and burden for factors and/or comorbidities (e.g., obesity, hypertension, advanced age). We added it in the paper (Lines 391 - 395). Thank you for this comment.

**Reviewer #2**: Thank you for the opportunity to review this interesting paper.

Am I correct in understanding that this study sampled only patients in inpatient settings? This limits the generalizability of the results presented here. On that note, I would ask why foreigners were excluded? And, how were subjects selected in the facility?

**Reply**: Dear colleague, yes you are right regarding inpatient setting. We updated the limitation section (Line 469). We excluded foreigners as not settling permanently in Douala, as outlined in the eligibility criteria. We enrolled participants consecutively using random sampling (Lines 108 - 110).

Re the sample size estimate: Sample size was chosen to ensure a precision of +/-5%. This is a relatively high error rate, especially when facility specific estimates are also given, which must have much larger error rates. For example, if there are 21 positive cases out of 22 subjects, the 95% CI extends from approximately 78.2% to 99.9%. For that reason, I'm not sure how useful it is to give facility specific estimates (and if that was the goal, the sample size should have been much higher in each facility).

**Reply**: We used a popular formula used to compute sample size for epidemiological studies. We used commonly used values for precision (5%). On a statistical view you’re right, but on a clinical view it is still important to specify how sample size was computed using default parameters as proposed in this formula. Thus, we think we can keep this section in the paper for clinicians. Besides, we tried to enrol maximum of participants within the study period. Even sample sizes are low for each health facility, we think it yet important to depict geographical variation of COVID-19 seroprevalence in our context, given the fact we noted that the bulk of patients were attending health facilities close to their home.

Please report the sensitivity and specificity of the diagnostic tests for IgG and IgM. The analysis strategy should account for test accuracy (since the probability of a positive test is not exactly the same as the probability of being seropositive), as discussed e.g. in Sempos and Tian 2020 DOI: 10.1093/aje/kwaa174.

**Reply**: Dear colleague, we agree with you. We added sensitivity and specificity values for IgG and IgM assays (Lines 150 - 152). We also added adjusted estimates of the seroprevalence in the paper as suggested even though no much difference was noted between crude and adjusted seroprevalence estimates (Lines 36 – 37, lines 176 – 183, lines 225 – 226).

Confidence intervals should be given for the seroprevalence estimates.

**Reply**: These intervals were given (Lines 223 – 225).

The time frame for this study was quite long at 9 months. Can you make any statement about seroprevalence at the beginning of the study vs at the end?

**Reply**: We added it some lines regarding this aspect (Lines 230 – 234).

Choice of variables in the multivariable models should be based on considerations related to e.g. confounding, not on univariable p-values. See Heinze and Dunkler 2017 DOI: 10.1111/tri.12895.

**Reply**: We agree with you. We updated multivariate logistic analysis, and rephrased where appropriate in the abstract (Lines 39 - 43), methods (Lines 190 - 191), results (Lines 316 - 328), discussion (Lines 422 - 429), and conclusion (Lines 484 - 485). We also added this paper by Heinze and Dunkler (2017). Thank you

Figure 3 would be much improved if confidence intervals could be incorporated.

**Reply**: We prefer keep this figure like this. But we added confidence intervals in the Supplementary file 3 (S3_Table R1).

In Table S5, I'm not sure sensitivity, specificity etc are used correctly. Please check this.

**Reply**: You’re right dear colleague. The sensitivity, specificity, predictive values were computed for IgM (proxy of active infection), not for IgG as previously presented in the Table S5 title. We corrected this mistake. Sorry for this inconvenience.

The link to the data on Dryad did not work for me.

**Reply**: You can also use this link that we added on line 525 (<https://datadryad.org/stash/share/F9asJatStkwKV-o9dMR-XP9-7N4KIARfRotfB-OMcyE>).

Thank you for your comments

Regards

Name of the corresponding author: **Prof. Koanga Mogtomo Martin Luther**

E-mail: : [koanga @yahoo.com](mailto:koanga@yahoo.com)

Phone: +237 699 503 444

Address: Department of Biochemistry, Faculty of Science, The University of Douala, P.O Box 24157, Littoral region, Cameroon
